# From a Movement-Deficient Grapevine Fanleaf Virus to the Identification of a New Viral Determinant of Nematode Transmission

**DOI:** 10.3390/v11121146

**Published:** 2019-12-11

**Authors:** Lorène Belval, Aurélie Marmonier, Corinne Schmitt-Keichinger, Sophie Gersch, Peggy Andret-Link, Véronique Komar, Emmanuelle Vigne, Olivier Lemaire, Christophe Ritzenthaler, Gérard Demangeat

**Affiliations:** 1 Université de Strasbourg, INRAE, SVQV UMR-A 1131, 68000 Colmar, France; lorene.belval@uha.fr (L.B.); aurelie.marmonier@inra.fr (A.M.); keichinger@unistra.fr (C.S.-K.); sophie.gersch@inra.fr (S.G.); veronique.komar@inra.fr (V.K.); emmanuelle.vigne@inra.fr (E.V.); olivier.lemaire@inra.fr (O.L.); 2 Université de Strasbourg, CNRS, IBMP UPR 2357, 67000 Strasbourg, France

**Keywords:** transmission, *Nepovirus*, grapevine, nematode, *Xiphinema*, capsid, 3D structure, movement, tubule, viral determinant

## Abstract

Grapevine fanleaf virus (GFLV) and arabis mosaic virus (ArMV) are nepoviruses responsible for grapevine degeneration. They are specifically transmitted from grapevine to grapevine by two distinct ectoparasitic dagger nematodes of the genus *Xiphinema*. GFLV and ArMV move from cell to cell as virions through tubules formed into plasmodesmata by the self-assembly of the viral movement protein. Five surface-exposed regions in the coat protein called R1 to R5, which differ between the two viruses, were previously defined and exchanged to test their involvement in virus transmission, leading to the identification of region R2 as a transmission determinant. Region R4 (amino acids 258 to 264) could not be tested in transmission due to its requirement for plant systemic infection. Here, we present a fine-tuning mutagenesis of the GFLV coat protein in and around region R4 that restored the virus movement and allowed its evaluation in transmission. We show that residues T258, M260, D261, and R301 play a crucial role in virus transmission, thus representing a new viral determinant of nematode transmission.

## 1. Introduction

Capsid proteins (CPs) not only protect the genome of plant viruses, but are often crucial in a variety of other functions including vector transmission, replication, translation, cell-to-cell and long distance movements, pathogenicity, inactivation of plant antiviral defenses, or host range determination [1]. CPs of plant viruses bear the determinants of essential interactions with vector and host factors or even with other virus proteins. For instance, CP determinants have been implicated in the interaction with the C-terminus of movement proteins (MPs) belonging to the 30K superfamily to ensure specific virus transport [2]. Such CP determinants are also involved in virus transmission, as exemplified with potyviruses [3,4] or cucumber mosaic virus (CMV) [5,6]. The highly-conserved motif of three amino acids, Asp-Ala-Gly (DAG motif) of potyviral CPs interacts with the HC-Pro helper component to ensure virus transmission [4,7]. Similarly, conserved negatively charged residues of the outer surface exposed βH-βI loop of the CMV capsid were shown to be essential for virus transmission by a given aphid species [6]. Additionally, residues involved in quaternary interactions of the CMV particle at the quasi-threefold axis of symmetry were involved in virus transmission [5]. These examples illustrate the importance of specific CP residues in both the movement and vector transmission of plant viruses.

Grapevine fanleaf virus (GFLV) and arabis mosaic virus (ArMV) are two plant viruses belonging to the subgroup A of the genus *Nepovirus* in the family *Secoviridae* [8]. They are the major pathogens responsible for infectious degeneration, one of the most severe viral diseases of grapevines worldwide [9,10]. The natural spread of GFLV and ArMV is specifically accomplished from grapevine to grapevine by two soil-borne ectoparasitic nematodes species, *Xiphinema index* and *X. diversicaudatum*, respectively. Nematodes transmit the virus while feeding on actively growing rootlets [11,12]. GFLV and ArMV genomes are composed of two positive-sense RNAs [13] and share a common organization. Each RNA is translated into a polyprotein that is cleaved in functional proteins [14]. RNA1 encodes proteins necessary for replication, processing of the polyproteins [15], and is involved in the symptomatology [16], whereas RNA2 encodes three proteins: the 2A^HP^ protein involved in RNA2 replication [17] and virus symptomatology [18]; the movement protein (2B^MP^) that self-assembles into tubules [19] (i.e., large macromolecular complexes embedded in plasmodesmata and enabling cell-to-cell movement of the virus as entire particles); and finally, the 2C^CP^ capsid protein [20].

Proteins 2C^CP^ of GFLV and ArMV (504 and 505 amino acids, respectively) are organized in three jelly-roll β sandwich domains commonly found in icosahedral capsids, called C, B, and A from the N- to the C-terminal end of the protein [21]. Sixty identical CP subunits self-assemble to produce a ca. 30 nm icosahedral capsid with a pseudo *T* = 3 symmetry [21]. This capsid ensures the virus genome protection [13], constitutes the entity moving from cell-to-cell and systemically in plants, and determines the virus transmission by the nematode vector. The cell-to-cell movement of the virus was shown to require specific interactions between the 2B^MP^ C-terminus and the 2C^CP^ [22]. The capsid was also demonstrated to bear the determinants for the specific retention of GFLV and ArMV in their respective soil-borne vectors [23,24,25,26]. Thus, GFLV and ArMV CPs must possess residues specialized in interactions devoted to these different processes.

In a previous work, we defined five amino acid regions called R1 to R5 in the GFLV-2C^CP^, based on their predicted exposition on the outer surface of the capsid, their conservation among the GFLV strains, and divergence between GFLV and ArMV. These regions represent good candidate motifs for the specific movement and/or transmission of GFLV [27]. By replacing regions R1 to R5 in the GFLV-2C^CP^ with their ArMV counterparts, we generated the chimeric viruses called G1 to G5 and identified a stretch of 11 residues in the βB-βC loop of domain B (region R2, residues 188 to 198) as a viral transmission determinant. We could further exclude region R1 (residues 79 to 85) from transmission specificity [27]. Regions R3 (residues 207 to 210) and R5 (residues 297 to 305) appeared to be involved in proper genome encapsidation and protection as deduced from the failure of the capsids of the chimeric constructs G3 and G5 to protect the genomic RNAs in an RNase protection assay [27]. Finally, the substitution of region R4 (residues 258 to 264) in the GFLV-2C^CP^ by its ArMV counterpart (G4 chimeric construct) led to viral RNA protection in protoplasts, but not to systemic spread of the chimeric virus [27]. From these results, we hypothesized that regions R3 and R5 were involved in capsid formation while region R4 could be implicated in specific tubule-capsid interactions needed for cell-to-cell and/or long-distance movement of the virus. Due to the defect of G4 to spread systemically, the involvement of region R4 in the transmission of GFLV could not be assessed. Region R4 presents a special interest as a transmission determinant because it lies in the vicinity of both region R2 and a single residue of region R5, which was shown to constitute a second viral determinant of GFLV transmission by *X. index* [21]. This single residue, Gly297, and region R2 together delineate a positively charged cavity, as deduced from the structure of GFLV that was obtained at a 3 Å resolution, and could serve as a binding pocket [21] for the retention of the virus within the nematode.

To test the involvement of region R4 in GFLV transmission, we conducted a site-directed mutagenesis to recover the movement of a chimeric GFLV harboring the ArMV R4 region. To this end, we introduced a sequence encoding Enhanced Green Fluorescent Protein (EGFP) into the GFLV genome in order to visualize the virus propagation. We then introduced point mutations in the recombinant G4-EGFP virus, in and around region R4, and could restore a fully infectious virus bearing point mutations in regions R4 and R5. This fine-tuning mutagenesis allowed uncoupling cell-to-cell from the long distance movement of the virus and identified a new determinant of GFLV transmission by its nematode vector *X. index*.

## 2. Materials and Methods

### 2.1. Virus Strains and cDNA Clones

GFLV strain F13 [28] and ArMV strain Syrah (ArMV-S) [29] were isolated from naturally infected grapevines and propagated in *Chenopodium quinoa*, a systemic host for both viruses. Full-length infectious cDNA clones of GFLV-F13 were previously described [30]. The ArMV-S RNA2-U sequence was used to generate the GFLV/ArMV chimeric CP sequences [21,27]. ArMV-Co, a local ArMV isolate exclusively transmitted by *X. diversicaudatum*, was used as a negative control in *X. index* transmission assays (GenBank accession nos. MN599984 and MN599985 for RNA1 and 2, respectively).

### 2.2. Cloning of 2C^CP^ Mutations into GFLV-EGFP Infectious Clones

Plasmid pVecP2-2A:EG contains the cDNA of GFLV-RNA2 and the EGFP coding sequence inserted between the 2A^HP^ and 2B^MP^ coding sequences [31]. This plasmid codes for a suboptimal R/G cleavage site between the 2A^HP^ and EGFP domains within the polyprotein P2 and allows the expression of a 2A:EGFP fusion protein in addition to free EGFP (see also Figure 2).

To produce the chimeric G3-EGFP and G4-EGFP infectious RNA2 clones, the *Bam*HI/*Xma*I restriction fragment of plasmids pVec_Acc65I_2ABC_G3_, and pVec_Acc65I_2ABC_G4_ [27] (see also Figure 2) was subcloned into pVecP2-2A:EG. The ∆CP-EGFP cDNA clone was obtained by introducing the *Age*I/*Sal*I restriction fragment of plasmid pVec2AB [17] into pVecP2-2A:EG.

Site directed mutagenesis within the R4 region was performed using the QuickChange II kit (Agilent, Stratagene, Santa Clara, CA, USA) following the supplier’s instructions and the oligonucleotide primers listed in Table S1. Mutants E264A-EGFP, E264D-EGFP, E264Q-EGFP, TMD_Ala-EGFP, and TMD_ArMV-EGFP were generated in two steps. First, mutations were introduced in the plasmid pVec_Acc65I_2ABC, corresponding to the GFLV RNA2 wild type template [27], then, the *Bam*HI/*Xma*I restriction fragments of the mutant constructs were subcloned into the EGFP coding pVecP2-2A:EG. To produce mutants TMDR_Ala-EGFP and TMDR_ArMV-EGFP, residue R301 was first substituted by an alanine or a serine (ArMV equivalent residue), respectively, in the wild type template pVec_Acc65I_2ABC. The *Xma*I/*Bgl*II restriction fragment bearing the R301A or R301S substitution was then subcloned into the mutant plasmids TMD_Ala-EGFP and TMD_ArMV-EGFP to generate the mutants TMDR_Ala-EGFP and TMDR_ArMV-EGFP, respectively (see also Figure 2).

To produce the chimeric RNA2 TMDR_ArMV devoid of the EGFP sequence, the *Bam*HI/*Bgl*II restriction fragment from the construct TMDR_ArMV-EGFP was subcloned in the pVec_Acc65I_2ABC plasmid.

### 2.3. Capsid Structure Representation and Analyses

Capsid representations were made using the previously 3 Å resolved GFLV-F13 atomic structure (PDB ID: 4V5T) [21] with the UCSF Chimera package [32].

### 2.4. In Vitro Transcription and Inoculation

GFLV-F13 RNA1 and GFLV wild-type or modified RNA2 transcripts were obtained from corresponding cDNA clones by in vitro transcription with the mMESSAGE mMACHINE T7 kit (Ambion, Thermo Fisher Scientific, Illkirch-Graffenstaden, France) after linearization with *Bgl*II and *Sal*I, respectively [16]. *C. quinoa* plants were mechanically inoculated with transcripts of GFLV RNA1 and RNA2 as described in [30]. In the inoculated leaves, EGFP fluorescence was monitored at seven days post inoculation (dpi) to visualize the cell-to-cell movement. Systemic infection was assessed in uninoculated apical leaves at two to three weeks post inoculation by symptom or fluorescence observations and DAS-ELISA.

### 2.5. Fluorescence Visualization and Data Processing

Confocal observations were performed with a Zeiss LSM-510 inverted laser scanning confocal microscope equipped with a Kr/Ar laser. Images were processed with LSM5 (Carl Zeiss AG, Oberkochen, Germany) or ImageJ (http://imagej.nih.gov/ij/) software.

Low magnification fluorescence imaging of infected leaves was performed with a Zeiss Axio-Zoom V16 stereomicroscope equipped with the apochromatically corrected zoom system Z16 APO and a 5X objective. EGFP visualization was obtained by epifluorescence excitation and emission of 450–490 nm and 500–550 nm, respectively. Pictures were taken with a DFC 360FX camera under identical illumination and exposure conditions to allow comparison between samples. For some experiments, low magnification fluorescence imaging was realized with a Nikon E400 epifluorescence microscope equipped with a 4X objective. EGFP visualization was obtained with an excitation and emission of 480–540 nm and 535–550 nm, respectively. Fluorescent foci on pictures were delineated and their surfaces were measured using ImageJ software. GFLV-EGFP served as a reference as it expresses a wild type GFLV-2C^CP^. Analysis and representation were performed using the XLSTAT software version 2015 (Addinsoft, Paris, France). The global significant difference between sample distribution functions was first confirmed using the Kruskal–Wallis test (bilateral *p*-value < 0.0001). Specific differences between modalities were then assessed using the post-hoc Steel–Dwass–Critchlow–Fligner distribution-free multiple comparison test. This non-parametric test is used to perform all possible pairwise comparisons between conditions, from which differences were considered significant at a level of *p* < 0.03.

### 2.6. Virus-Like Particle Production in Plants

Expression vectors were obtained by Gateway cloning: the sequence of the chimeric or mutated 2C^CP^s were PCR amplified using pVecP2 derived plasmids as templates and primers harboring the attB1 and attB2 sequences. Amplicons were first recombined into the pDONR/Zeo vector and further recombined into the plasmid pEAQ-HT-DEST1 binary plasmid [33]. For the transient expression of CPs C-terminally tagged with TagRFP (TR) [34], pDONR/Zeo entry vectors were constructed that contained mutated CP sequences devoid of the stop codon. They were recombined into the expression vector pEAQgwTR [35]. Recombination resulted in the introduction of the DPAFLYKVVRSFGPA linker peptide between the 2C^CP^ and TR proteins. All plasmids were sequenced and transferred into *Agrobacterium tumefaciens* GV3101 pMP90 [36] by electroporation.

Virus-like particles (VLPs) were produced by the transient expression of the 2C^CP^ after agroinfiltration of *Nicotiana benthamiana* leaves as described [37], except that the bacterial suspension was infiltrated in three-week-old plants five days before leaf harvesting.

### 2.7. Double-Antibody Sandwich-Enzyme-Linked Immunosorbent Assay (DAS-ELISA)

Virus and VLP production were analyzed by DAS-ELISA from mechanically inoculated *C. quinoa* or agro-infiltrated *N. benthamiana* leaves. Plant material was ground at 1:5 w/v ratio in 25 mM sodium phosphate buffer (pH 7.4) and clarified by 5 min centrifugation at 4000 g. GFLV virions or VLPs were detected using a commercial kit (Bioreba, Reinach, Switzerland) following the manufacturer’s instructions. Absorbance of the hydrolyzed substrate (*para*-nitrophenylphosphate) was measured at 405 nm with a Titertek Multiscan MCC/340 reader (Labsystems, Helsinki, Finland).

### 2.8. Immunocapture Reverse Transcription Polymerase Chain Reaction (IC-RT-PCR) and Sequencing

GFLV particles were immunocaptured from plant extracts in microtiter plates precoated with polyclonal anti-GFLV antibodies (Bioreba). RT-PCR was designed to amplify three overlapping cDNA fragments F1, F2, F3 that covered the last 70% of the 2B^MP^ coding sequence, the complete 2C^CP^ coding sequence, and the 3′ untranslated region using RNA2 specific primers (Table S2). More precisely, the three fragments F1, F2, F3 overlap the 2B^MP^, 2C^CP^, and 3′UTR sequences from the nucleotides 1319 to 3774 of GFLV-F13 RNA2 (GenBank accession no. NC_003623; see also Figure 2). The DNA fragments were analyzed by electrophoresis in an agarose gel and sequenced with appropriate primer pairs (Table S2). Nucleotide sequences were analyzed using the Vector NTI software (InforMax, Life technologies, Thermo Fisher Scientific, Carlsbad, CA, USA).

### 2.9. Electron Microscopy

Healthy, infected, or agro-infiltrated leaves were ground in 25 mM sodium phosphate buffer pH 7.4 and clarified at 4000 *g* for 5 min at 4°C. The 300 mesh formvar carbon-coated nickel grids were coated with polyclonal anti-GFLV antibodies and covered with clarified plant extracts. Following a saturation step with BSA 2% w/v, normal goat serum 10% v/v, Triton X100 0.05%, and 22.5 mM sodium phosphate buffer pH 7.4, the grids were further incubated with a mix of three homemade monoclonal anti-GFLV antibodies [17]. Immunogold labelling was performed using anti-mouse antibodies conjugated with 10 nm gold particles (Sigma-Aldrich, Saint-Louis, MO, USA). Three washes with sodium phosphate buffer preceded each step of the decoration procedure. Samples were negatively stained with a 1% ammonium molybdate solution prepared in the grinding buffer. For further analysis, a fixation step was performed before saturation, using a solution of 1% paraformaldehyde diluted in the grinding buffer. Observations were done on a Philips EM208 transmission electron microscope. Film-based photographs were acquired onto Kodak Electron Image Films SO-163 and developed. Photographs were scanned to obtain digital images.

### 2.10. Western Blot Analysis

For each sample, three discs 1 cm in diameter were collected from agro-infiltrated *N. benthamiana* leaf patches, five days post infiltration (dpi), ground in 270 µL of phosphate buffered saline (pH 7.4) in the presence of protease inhibitors (cOmplete™: Roche Diagnostics GmbH, Mannheim, Germany), and mixed with 1 volume (w/v) of Laemmli buffer 2X [38]. Total soluble proteins were heat-denatured 10 min at 90 °C and clarified at 10,000 g for 5 min prior to separation on an 8% acrylamide SDS-PAGE (sodium dodecyl sulfate-polyacrylamide gel electrophoresis). Resolved proteins were electro-transferred onto Immobilon PVDF (Polyvinylidene Difluoride) membranes and incubated with commercial polyclonal anti-TagRFP antibodies (Evrogen, Moscow, Russia) at a 1:5000 dilution. The detection step was achieved with goat anti-rabbit antibodies conjugated to horseradish peroxydase at a 1:12,500 dilution (Life technologies, Thermo Fisher Scientific) and with the Lumi-Light chemiluminescence system (Roche Diagnostics GmbH). The membrane was then imaged with the G:Box system (Syngene, Cambridge, UK).

### 2.11. Nematode Transmission Tests

The nematode transmission tests were performed under greenhouse conditions using aviruliferous *X. index* nematodes isolated from rearings established on fig plants (*Ficus carica*), and using a two-step procedure including an acquisition access period (AAP) and an inoculation access period (IAP) [24,27]. Two independent transmission experiments (Exp1 and Exp2) were carried out. For Exp1, about 350 nematodes fed on an infected *N. benthamiana* source plant grown in 0.5 L plastic pots. After an AAP of six weeks, each infected source plant was replaced by a healthy *N. benthamiana* bait plant and grown in greenhouse for an IAP of six weeks. In Exp2, about 19,000 *X. index* nematodes were fed on 12 infected source plants grown in a single 9 L container. After, an AAP of six weeks, the soil was collected and groups of ca. 300 *X. index* nematodes from the AAP step were deposited around the roots of individual healthy *N. benthamiana* bait plants in 0.5 L plastic pots for an IAP of eight weeks. In both procedures, the nematode transmission of the virus was assessed in the roots of bait plants by DAS-ELISA [24,27]. The access of the vector to viruses was verified in nematodes by RT-PCR after the AAP as described in [25].

## 3. Results

### 3.1. Structural Environments of Regions R3, R4, and R5 of the GFLV 2C^CP^

**Figure 1:**
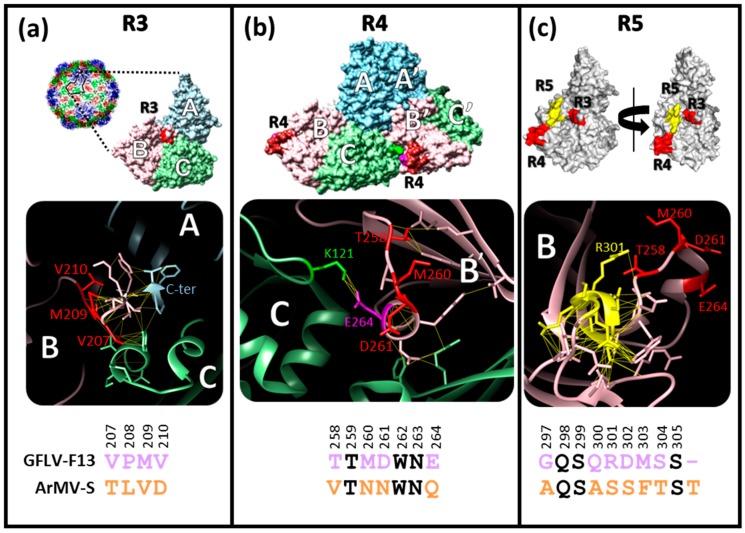
Structural features of regions R3, R4, and R5 in the GFLV-F13 2C^CP^. Each panel is divided in three sections. From top to bottom: surface view(s) of the grapevine fanleaf virus (GFLV) subunit calculated from the GFLV atomic structure (PDB: 4V5T), a close-up view of the targeted region in a ribbon representation and the amino acid sequence comparison of the region between GFLV-F13 and ArMV-S. In the two first panels, the A, B, and C jelly-roll domains of each subunit are colored in light blue, pink, and green, respectively. (**a**) R3 maps to the center of a capsid subunit at the junction of the three jelly-roll domains. (**b**) R4 is shown on two neighboring GFLV subunits with the three jelly-roll domains of the two subunits named A to C and A’ to C’, respectively. (**c**) Surface view with a lateral anti-clockwise rotation of 90 °C. R3 and R4 regions are depicted in red and R5 in yellow. In the middle sections, amino acids involved in contacts are represented as stick models to view the lateral chains. The putative non-covalent bonds between neighboring residues are illustrated with thin yellow interatomic segments for d < 3 Å. Sequence alignments were created using AlignX (Vector NTI, InforMax). Positions within the 2C^CP^ sequence are given above each amino acid according to the F13 reference. The purple line at the end of GFLV region R5 (panel c, bottom section) represents a gap in the sequence alignment. Specific GFLV and ArMV residues are shown in purple and orange, respectively, whereas conserved residues are in black.

Regions R3 (residues 207 to 210), R4 (residues 258 to 264), and R5 (residues 297 to 305) located in the jelly-roll domain B of the 2C^CP^ protein were initially predicted to be exposed at the outer surface of the GFLV capsid from a GFLV 3D homology model derived from TRSV [27]. These regions differ between GFLV and ArMV and are described as motifs with possible function in encapsidation, movement, and transmission of GFLV. Following the obtention of the GFLV-F13 atomic structure at 3 Å resolution [21], we refined the structural features of these regions (Figure 1).

The four residues of region R3 (207-VPMV-210) are all exposed at the outer surface of the capsid, in the central zone of the capsid subunit at the intersection of the three jelly-roll domains (A, B, and C), and at the vicinity of the last C-terminal residues of the protein (Figure 1). These four residues are involved in a dense network of interactions with their neighboring residues (highlighted by yellow interatomic segments for d < 3 Å in Figure 1) including the C-terminus of the 2C^CP^ chain, suggesting that these four residues play a crucial role in the conformation of the capsid subunit (Figure 1). In addition, the P208 residue located in region R3 likely confers an essential conformational rigidity to the 2C^CP^ backbone. Altogether, these observations explain the importance of region R3 for the global structure and stability of the capsid (Figure 1) and justify the encapsidation defect observed for the chimeric virus G3 [27].

Region R4 corresponds to the amino acid stretch 258-TTMDWNE-264 where four residues (T258, M260, D261, and E264) differ between GFLV and ArMV. This region is located at the edge of the capsid subunit and is in close contact with the C domain of the neighboring subunit (Figure 1). Therefore, this region is likely to play a critical role in CP/CP interactions. Among the four divergent GFLV/ArMV residues, E264 is buried in the GFLV-F13 structure and makes a salt bridge with residue K121 of the neighboring CP subunit. The presence of such a strong interaction suggests a key role of this E/K interaction in the stability of the capsid (Figure 1). The GFLV atomic structure also indicates that T258 is exposed at the outer surface and is close to the next CP subunit in the pentamer (icosahedral 5-fold axis). D261 is not directly involved in CP/CP interactions but contacts other side chains of the same subunit. M260 is pointing toward the solvent and is free of interaction. The other R4 residues conserved between GFLV and ArMV are not exposed to the outer surface of the CP, but are involved in the CP local folding or directly in CP–CP interactions within the pentamer.

The GFLV structure also highlights the remarkable residue R301 of region R5 that is conserved among GFLV isolates, divergent from ArMV (S301), and well outer surface exposed. Unlike most of the other residues of region R5 that are buried or involved in tertiary or quaternary CP/CP interactions, residue R301 is exposed at the outer surface and points toward T258 and M260 of the region R4 (Figure 1).

In the light of the GFLV atomic structure and according to the already known biological properties of recombinant viruses G3, G4, and G5 [27], we evaluated the four divergent residues T258, M260, D261, E264, of region R4 for their involvement in virus movement and transmission and extended our mutagenesis strategy to R301 of region R5.

### 3.2. A GFLV Encoding EGFP Allows Short Distance Movement Visualization in Planta

**Figure 2:**
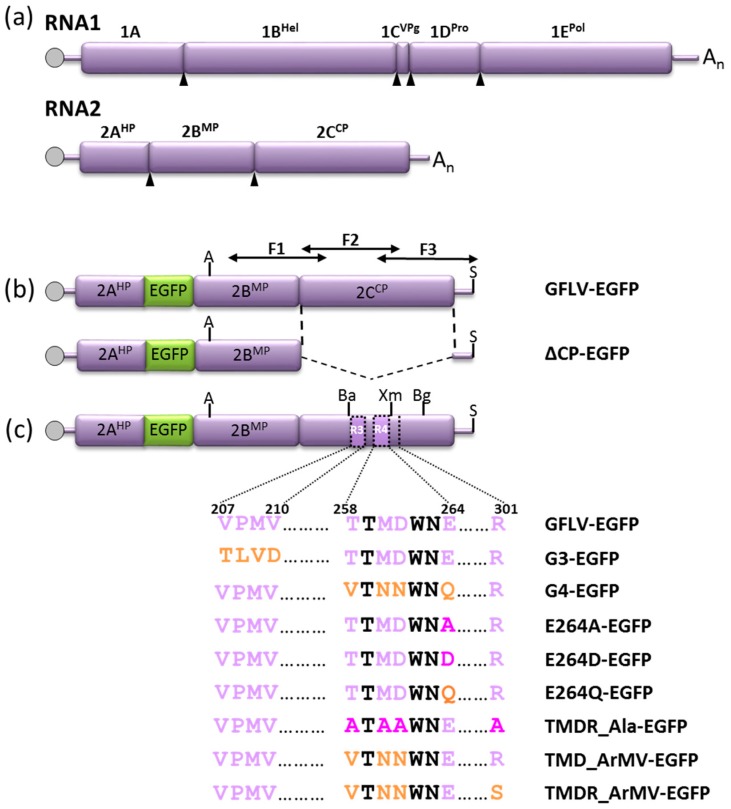
Genetic organization of GFLV-F13 RNA1 and RNA2 (**a**) and schematic representation of GFLV-F13 recombinant RNA2 used in this study (**b**,**c**). Open purple boxes indicate open reading frames. The 5′ and 3′ untranslated regions (UTRs) are denoted by a purple thick line and the VPg is represented by a gray circle. Arrowheads point to the polyprotein cleavage sites. The name of the processed proteins is given above the boxes: Hel = putative helicase, VPg = viral protein genome linked, Pro = protease, Pol = polymerase, HP = putative homing protein, MP = movement protein, and CP = coat protein. (**a**) Wild type RNA1 and RNA2 of GFLV-F13. (**b**) Recombinant wild type (RNA2-EGFP), and CP deleted (∆CP-EGFP) RNA2 expressing both a 2A:EGFP fusion protein and some free EGFP. Black arrows denoted F1, F2, and F3 show the position of the three DNA fragments amplified by IC-RT-PCR to sequence the virus progeny. (**c**) Recombinant GFLV-EGFP RNA2 expressing mutated CPs, with a focus on amino acids from regions R3 and R4 and in position 301. Residues of GFLV origin are in purple, residues of ArMV origin are in orange, and conserved residues are in black. Pink residues are neither of GFLV nor of ArMV origin. Restriction sites used for cloning purpose are positioned; Xm = *Xma*I (nts 2852–2857, GenBank accession no. NC_003623); Bg = *Bgl*II (nts 3055–3060), Ba = *Bam*HI (nts 2299–2304), A = *Age*I (nts 1019–1024), and S = *Sal*I (immediately downstream of the 3’UTR sequence in the cDNA infectious clone).

In order to visualize GFLV cell-to-cell movement in planta, we used an EGFP encoding cDNA infectious clone of GFLV-RNA2 [31] along with the infectious clone of GFLV-RNA1 [30] to generate the GFLV-EGFP inoculum (Figure 2). A movement-deficient control was obtained by deleting the 2C^CP^ coding sequence from the recombinant EGFP encoding infectious clone (∆CP-EGFP construct) (Figure 2). The in vitro transcripts derived from these cDNA clones were inoculated to *C. quinoa* plants and inoculated leaves were assessed for the development of fluorescent foci.

**Figure 3:**
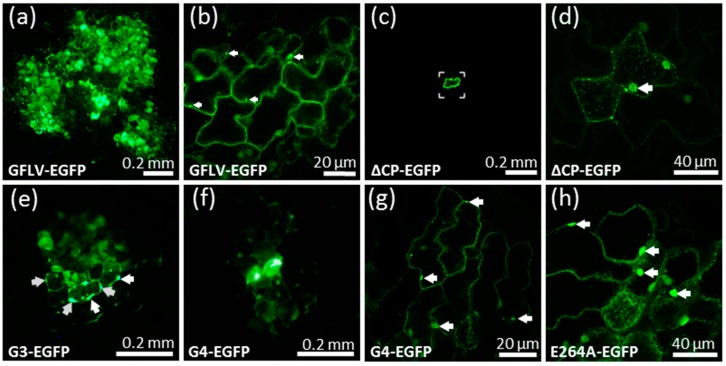
Cell-to-cell movement of synthetic GFLV viruses harboring a wild type or mutated 2C^CP^. *C. quinoa* plants were inoculated with in vitro transcripts of GFLV RNA1 and an EGFP expressing RNA2: (**a,b**) GFLV-EGFP (wild type control), (**c**,**d**) ∆CP-EGFP; (**e**) G3-EGFP, (**f**,**g**) G4-EGFP, or (**h**) E264A-EGFP. At 7 dpi, the inoculated leaves were observed with a stereomicroscope (**a**,**c**,**e**,**f**) or with a confocal microscope (**b**,**d**,**g**,**h**). GFLV-EGFP and G4-EGFP (**b**,**h**) are confocal images whereas ΔCP-EGFP and E264A-EGFP (**d**,**h**) are Z stacks. White arrows point to viral replication factories.

**Figure 4:**
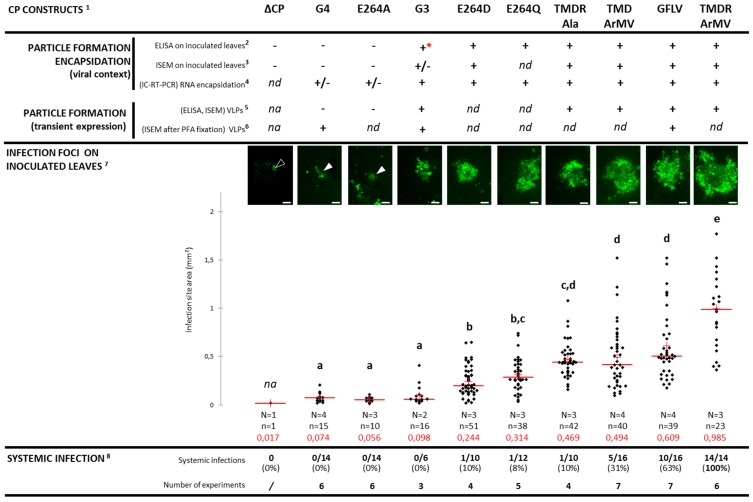
Effect of 2C^CP^ mutations on cell-to-cell movement, particle formation, RNA encapsidation, and systemic infection. ^1^ All mutants encode the EGFP to visualize the cell-to-cell movement (See Figure 2 for the description of the 2C^CP^ mutants). ^2^ GFLV DAS-ELISA performed on inoculated leaves at 7 dpi. Sample considered positive (+) when the A_405nm_ value exceeded the healthy control sample mean value by at least a factor of three. * A_405nm_ value of G3-EGFP is only three times the healthy value (see also Figure 5). ^3^ Immunosorbent electron microscopy (ISEM) performed on crude extracts from inoculated leaves used in DAS-ELISA at 7 dpi. (+) corresponds to observation of captured viral particles. (+/−) indicates that the capsid observations were very rare and barely noticeable (see also Figure 5). ^4^ Immuno-capture RT PCR (IC-RT-PCR) performed on crude extracts from inoculated leaves used in DAS-ELISA. (+) indicates the amplification of the three specific fragments covering the 2B^MP^ and 2C^CP^ coding sequences of RNA2. (+/−) corresponds to a clear amplification of only one of the three specific fragments. Fragment positions are indicated in Figure 2. ^5,6^ Transient expression of wild type and mutated 2C^CP^s in *N. benthamiana* leaves. Agro-infiltrated leaves were monitored for the 2C^CP^ expression by DAS-ELISA and by ISEM at seven days post agro-infiltration. ^6^ Agro-infiltrated crude extracts were fixed with 1% paraformaldehyde prior to observation by transmission electron microscopy. ^7^ Representative images and scattergram representations of the infection *foci* recorded at 7 dpi in *C. quinoa* inoculated leaves, for each 2C^CP^ mutant in two to four experiments. The empty arrowhead indicates a single cell while the white arrowheads indicate very small foci. Scale bars represent 0.2 mm. N = number of independent experiments. n = number of measured foci. The red cross and the red number in each graph represent the mean value and the horizontal bar corresponds to the median value. Different letters (a, b, c, d, e) above the graph indicate statistically significant differences between the different 2C^CP^ mutants determined by the Kruskal–Wallis test (bilateral *p*-value < 0.0001) and using the post hoc Steel–Dwass–Critchlow–Fligner bilateral test (*p* < 0.003 for all, except for the significant difference observed between GFLV-EGFP and TMDR_ArMV-EGFP at *p* = 0.027). ^8^ Data represent the number of symptomatic plants that reacted positively for GFLV in DAS-ELISA on systemic leaves at 21 dpi over the total number of plants analyzed in three to seven experiments. nd: not done; na: not applicable.

Seven days post inoculation (dpi), GFLV-EGFP induced the formation of numerous multicellular fluorescent foci on inoculated *C. quinoa* leaves. The green fluorescence was observed in small punctate structures and large aggregates (Figure 3, Figure 4). This pattern of fluorescence is in agreement with the previously described fate of protein 2A:EGFP during the course of protoplast infection and reflects its association with the viral replication factories [17,18]. On the contrary, only individual fluorescent cells were observed at 7 dpi when the leaves were inoculated with the ∆CP-EGFP transcripts (Figure 3). Occasionally, a weak and diffuse EGFP signal could be observed in the adjacent cells, likely reflecting the diffusion of free EGFP. However, no viral replication factory was visible in these neighboring cells and the fluorescence intensity never reached the level of fluorescence seen in the central cell (Figure 3), well in accordance with the absence of viral multiplication in the neighboring cells. After ten days, the fluorescence of recombinant virus GFLV-EGFP started to be visible in the apical non-inoculated leaves, which also began to show symptoms. This systemic infection was further confirmed by positive DAS-ELISA (Figure 4). As expected, plants inoculated with ∆CP-EGFP transcripts never showed systemic fluorescence, remained symptomless, and tested negative in DAS-ELISA (Figure 4) [17,22]. Altogether, these results indicate that the use of EGFP is a good means to assess the cell-to-cell movement of the virus.

### 3.3. Cell-to-Cell Movement of Chimeric Viruses G3-EGFP and G4-EGFP Is Impaired But Not Abolished

To test our previous hypothesis that chimeric virus G4 was impaired in cell-to-cell movement [27], we introduced its 2C^CP^ coding sequence in the GFLV-EGFP infectious clone to give the G4-EGFP construct (Figure 2). Chimeric virus G3 that encodes a 2C^CP^ protein, but does not protect its genomic RNAs from degradation [27] was considered as an appropriate encapsidation deficient control. Therefore, its 2C^CP^ coding sequence was also subcloned into the GFLV-EGFP construct to give the virus G3-EGFP (Figure 2).

Surprisingly, the chimeric construct G3-EGFP, unlike the ∆CP-EGFP control, was not restricted to single cells since numerous adjacent epidermal cells contained viral replication factories as seen from the fluorescent aggregates (Figure 3). However, the mean size of the fluorescent foci in the G3-EGFP inoculated leaves was drastically reduced when compared to those produced upon infection with the wild type GFLV-EGFP (Figure 4). Thus, a modified 2C^CP^ is compatible with some sort of cell-to-cell movement even when it is not fully effective in RNA protection, whereas its deletion completely abolishes the movement of GFLV. Chimeric construct G4-EGFP behaved like G3-EGFP as seen from the formation of fluorescent foci that are comparable in size to those formed with G3-EGFP, and much reduced when compared to the wild type GFLV-EGFP (Figure 4). The fact that cell-to-cell movement is possible for the chimeric construct G4-EGFP, similar to the encapsidation-hindered control G3-EGFP, suggests that encapsidation, rather that the movement *per se*, could be affected in the G4-EGFP virus despite its capacity to protect its genome in an RNase protection assay in protoplasts [27]. The monitored plants never showed symptoms in the upper non-inoculated leaves when G3-EGFP or G4-EGFP were used as the inoculum, whereas systemic leaves of plants infected with GFLV-EGFP showed typical mosaic symptoms (Figure 4). Negative DAS-ELISA confirmed the absence of systemic spread of G3-EGFP and G4-EGFP, thus supporting previous results [27].

### 3.4. Chimeric Virus G4-EGFP Like G3-EGFP Fails to Produce Stable Capsids

**Figure 5:**
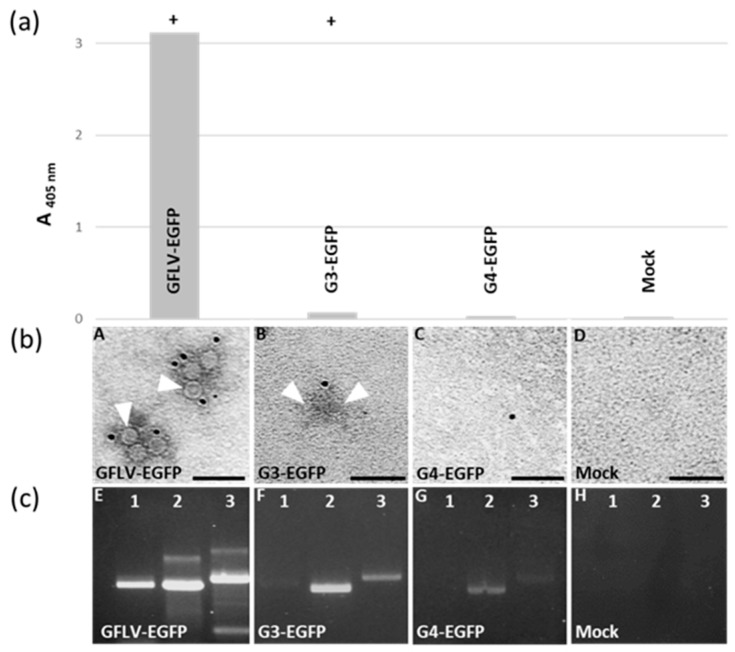
Particle formation and RNA encapsidation of GFLV chimeric viruses expressing the G3 2C^CP^ or the G4 2C^CP^. (**a**) *C. quinoa* infected leaves were analyzed at 7 dpi by DAS-ELISA, (**b**) ISEM, and (**c**) IC-RT-PCR. *C. quinoa* leaves were rubbed with in vitro transcripts of RNA1 and of GFLV-EGFP (**A,E**), G3-EGFP (**B**,**F**) or G4-EGFP (**C**,**G**) RNA2. (**a**) Bars represent the mean absorbance (A_405nm_) obtained with four different leaves for each condition. DAS-ELISA was considered positive (+) when it exceeded the healthy control value by at least a factor of three. (**b**) ISEM performed on the same crude extracts then analyzed by DAS-ELISA. Particles of 30 nm in diameter (white arrowheads) were detected in the GFLV-EGFP infected sample, but rarely and hardly in the G3-EGFP infected leaves. Scale bars: 100 nm. (**c**) Agarose gel analysis of the F1 (1029 bp, lane 1), F2 (1009 bp, lane 2), and F3 (1180 bp, lane 3) IC-RT-PCR fragments obtained from crude extracts used in DAS-ELISA.

The reduced cell-to-cell movement and absence of systemic spread of G4-EGFP are thought to result from a capsid malformation, like previously suggested for the chimeric virus G3 [27], although a reduction in the virus movement cannot be ruled out. To address the capacity of the G4-2C^CP^ to assemble fully-functional virions, we tried to detect G4 capsids from inoculated leaves (Figure 4, Figure 5). Based on our long-term experience, DAS-ELISA against GFLV gives positive results on native virus or virus-containing samples, but not on free 2C^CP^ subunits. This is exemplified in the G3-EGFP infected control that gives a drastically reduced signal (close to the positive threshold) when compared to the high values obtained with the wild-type GFLV-EGFP control (Figure 4, Figure 5). G4-EGFP recombinant tested negative, also suggesting an encapsidation-hindered behavior. Immunosorbent electron microscopy (ISEM) experiments allow the immunocapture of capsids from a crude leaf extract that are then immuno-labelled with antibodies conjugated to gold particles. Samples from the GFLV-EGFP inoculated leaves showed numerous capsids, whereas very rare and barely noticeable particles were observed with recombinant virus G3-EGFP samples and no particles were seen in G4-EGFP inoculated leaves (Figure 5). In an attempt to reveal genomic RNAs contained in very rare particles, immuno-capture reverse transcription PCR (IC-RT-PCR) experiments were performed on crude extracts from leaves inoculated by G3-EGFP, G4-EGFP, and GFLV-EGFP. PCRs were designed to amplify three genomic fragments, noted as F1, F2, and F3 covering the 3’ half of RNA2 (Figure 2). As expected, all three fragments were readily amplified from GFLV-EGFP infected leaves. Some amplification was also obtained from G3-EGFP and G4-EGFP inoculated samples although fragment F1 was less amplified from the G3-EGFP samples and not amplified from the G4-EGFP samples from which only F2 and F3 were poorly amplified (Figure 5) [27]. The sequence of fragment F2 from recombinant virus G4-EGFP was confirmed. These results suggest that the G4-2C^CP^ protein can, to some extent, protect viral RNA although it seems defective in proper capsid assembly.

**Figure 6:**
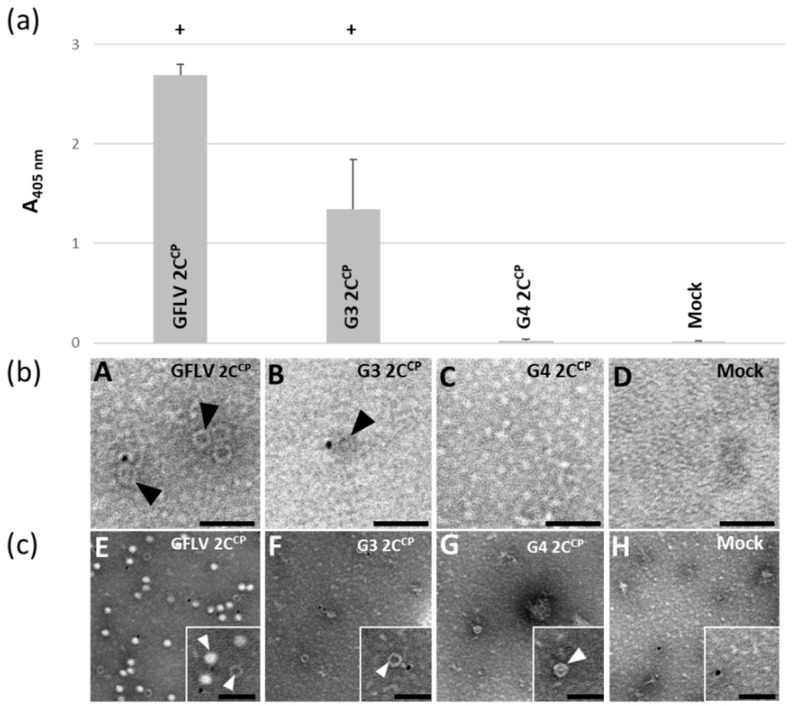
In planta ectopic expression of the chimeric G3 2C^CP^ and G4 2C^CP^. (**A**,**E**) GFLV 2C^CP^, (**B,F**) G3 2C^CP^, and (**C**,**G**) G4 2C^CP^ constructs were transiently expressed in *N. benthamiana* leaves. Agro-infiltrated leaves were analyzed by (**a**) DAS-ELISA or (**b**,**c**) ISEM. (**a**) DAS-ELISA was performed using anti-GFLV antibodies and A_405nm_ value was considered positive (+) when it exceeded the healthy control value by at least a factor of three. Bars represent the mean absorbance obtained with three different leaves for each condition. Error-bars correspond to 95% confidence intervals. (**b**) ISEM micrographs of observations performed on the same extracts then analyzed by DAS-ELISA. (**c**) Same as in (**b**), except that the crude extracts were fixed with 1% paraformaldehyde (PFA) before decoration, negative staining, and observation. Squares underlined with a white line correspond to an enlargement of the same ISEM micrographs. White arrowheads point to VLPs trapped by anti-GFLV antibodies in clarified leaf extracts. VLPs assembled from G3 2C^CP^ or G4 2C^CP^ are only clearly visible after PFA fixation. Scale bars: 100 nm.

As G4-EGFP only multiplies in a few cells, we wanted to rule out that the lack of capsid detection was due to a low level of subunit accumulation. We thus subcloned the G4 2C^CP^ coding sequence into a binary plasmid for agrobacterium-based transient over expression in *N. benthamiana* leaves. Wild-type GFLV 2C^CP^ and G3 2C^CP^ constituted the positive and negative controls, respectively. As expected, the overexpression of the GFLV 2C^CP^ led to the production of virus-like particles (VLPs) visualized by ISEM and detected by DAS-ELISA (Figure 6) [37]. Again, few and barely noticeable G3 VLPs were observed by ISEM, and their detection by DAS-ELISA was reduced when compared to the wild-type VLPs (Figure 6). In contrast, no G4 VLPs were visible by ISEM or DAS-ELISA in leaves agro-infiltrated with the G4 2C^CP^ expressing construct (Figure 6). When we fixed the agro-infiltrated leaves with paraformaldehyde before immunolabeling, G3 VLPs became clearly visible, but remained less numerous than wild-type VLPs and some G4 VLPs were very infrequently observed (Figure 6).

To check that the poor detection of G4 capsids is not due to a poor accumulation of the 2C^CP^ subunits in the agro-infiltrated leaves, and because none of the available anti-GFLV antibodies is able to detect the GFLV 2C^CP^ subunits in a western blot, we expressed the G4 2C^CP^ protein fused to a tag. Indeed, a C-terminal fusion of the 2C^CP^ to the fluorescent TagRFP (TR) was shown to be compatible with the production of VLPs [37]. When over-expressed in *N. benthamiana* leaves, the fusion protein G4 2C^CP^:TR was detected by anti-TagRFP antibodies in western blot (Figure S1) and remained undetectable by GFLV DAS-ELISA. These data demonstrate that chimeric G4 2C^CP^:TR subunits are produced in agro-infiltrated leaves and further confirm that DAS-ELISA allows for the discrimination of assembled capsids from non-assembled subunits.

Altogether, these results demonstrate that the G4 2C^CP^, like the encapsidation defective G3 2C^CP^, assemble in unstable capsids that are able to protect the genomic RNAs in an RNase protection assay [27], and allow a reduced cell-to-cell movement, but are unable to support a systemic infection. Our results also suggest that the substitution of region R4 by its ArMV counterpart has a more dramatic impact on the capsid stability than the substitution of region R3.

### 3.5. E264 Is Critical for Capsid Formation

Within region R4, the GFLV atomic structure highlights residue E264 as a strategic residue in the architecture of the capsid (Figure 1). To test the importance of this residue in the capsid structure, this glutamic acid was substituted by an alanine (no lateral chain but a conserved backbone), by an aspartic acid (a shorter lateral chain but the charge is conserved), or by a glutamine residue (the lateral chain length is conserved, but not the charge), this latter being present in region R4 of ArMV and thus in recombinant G4 and G4-EGFP. The mutated 2C^CP^ sequences were introduced in the GFLV-EGFP genome to produce the mutant viruses E264A-EGFP, E264D-EGFP, and E264Q-EGFP in order to follow their cell-to-cell movement (Figure 2).

The point mutant E264A-EGFP exhibited a phenotype similar to that of recombinant virus G4-EGFP since it induced the formation of small sized foci extending over several cell layers, was undetectable in DAS-ELISA, only allowed amplification of the F2 fragment in IC-RT-PCR, and did not spread systemically (Figure 3, Figure 4, Figure S2). Additionally, the E264A 2C^CP^:TR accumulated in agro-infiltrated leaves but seemed unable to assemble into stable VLPs as judged from the positive anti-TR western blot and negative anti-GFLV DAS-ELISA (Figure S1). In contrast, the two mutant viruses E264D-EGFP and E624Q-EGFP led to fluorescent foci having sizes significantly larger than those produced by E264A-EGFP or by G4-EGFP, but slightly smaller than the GFLV-EGFP wild type control. These two mutants generated a positive DAS-ELISA signal and the three expected fragments (F1, F2, F3) were amplified by IC-RT-PCR similarly to GFLV-EGFP, showing that they produce proper capsids and move normally from cell-to-cell (Figure 4, Figure S2). However, despite their apparent functional capsids, mutants E264D-EGFP and E624Q-EGFP only exhibited 8 and 10% of systemic events, respectively. The mutations were conserved in the progeny and no compensatory modification was identified after IC-RT-PCR and sequencing performed on the crude sap of apical non-inoculated leaves (Figure 4). Whether this default in long distance infection is attributable to the capsid structure or to the movement *per se* remains unresolved. Remarkably, mutation E264Q does not have the same effect on particle formation, cell-to-cell movement, and systemic infection when present in a GFLV R4 context (point mutant E264Q-EGFP) or in an ArMV R4 context (recombinant G4-EGFP). This result further indicates that the nature of region R4 is of importance for capsid formation and in planta movement.

Due to the importance of E264 for proper particle formation and/or stability, likely due to the salt bridge it forms with residue K121 of the neighboring 2C^CP^ subunit (Figure 1), we decided to exclude it from the R4 swapping between GFLV and ArMV, aiming at generating an infectious chimera in region R4.

### 3.6. Generation of an Infectious Chimera in Region R4

In order to restore the systemic movement of a GFLV mutated in region R4, we analyzed the involvement of four additional residues: the three remaining residues of R4 that diverge between GFLV and ArMV (T258, M260, D261) and the spatially very close residue R301 belonging to region R5 (Figure 1). First, the three residues of region R4 were substituted by their ArMV counterparts to give the recombinant virus TMD_ArMV-EGFP (Figure 2). Then, residue R301 was added in the substitution to generate the recombinant virus TMDR_ArMV-EGFP. Finally, the mutant virus TMDR_Ala-EGFP, in which T258, M260, D261 and R301 are replaced by four alanines, was constructed (Figure 2). These three new mutant viruses were assessed for their cell-to-cell movement by the observation of fluorescent infection foci on inoculated leaves and their capacity to produce systemic infection.

**Figure 7:**
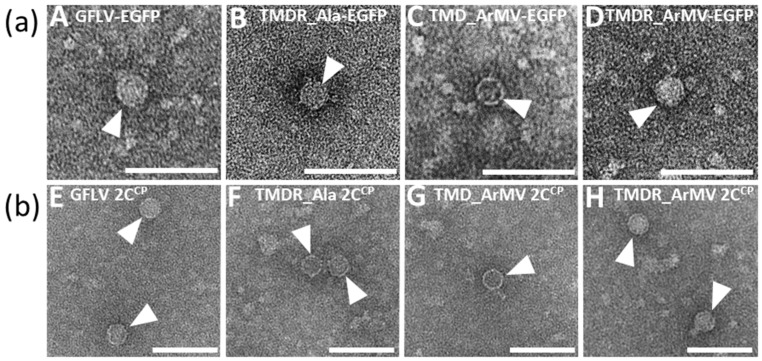
Capsid assembly of (**a**) mutant viruses or (**b**) transiently expressed capsid proteins. (**a**) ISEM analyses performed on crude extracts from *C. quinoa* leaves inoculated with RNA1 and an EGFP-expressing RNA2: (**A**) GFLV-EGFP, (**B**) TMDR_Ala-EGFP, (**C**) TMD_ArMV-EGFP or (**D**) TMDR_ArMV-EGFP. (**b**) ISEM performed on crude extracts of *N. benthamiana* leaves transiently expressing (**E**) the GFLV 2C^CP^, (**F**) the TMDR_Ala 2C^CP^, (**G**) the TMD_ArMV 2C^CP^, or (**H**) the TMDR_ArMV 2C^CP^. Crude extracts were loaded on grids precoated with anti-GFLV antibodies before being negatively stained. All viral particles looked well-formed and morphologically uniform. The scale bars indicate 100 nm.

The mutant virus TMDR_Ala-EGFP generated fluorescent foci at 7 dpi with a mean size equivalent to the wild type GFLV-EGFP. Capsid formation was confirmed by positive DAS-ELISA and successful capsid trapping from both infected leaves and agrobacterium-based transient expression of its 2C^CP^ (Figure 4, Figure 7). However, despite an efficient cell-to-cell movement and the detection of its RNA2 by IC-RT-PCR, suggesting a correct encapsidation of its genomic RNAs, this mutant only rarely developed a systemic infection (Figure 4, Figure S2). Analysis of the virus progeny in the upper non-inoculated leaves of the single systemically infected plant revealed no reversion and no compensatory mutation. Altogether, these results demonstrate that residues T258, M260, D261, and R301 are not essential for cell-to-cell movement of the virus, but play an important role in the systemic movement, suggesting that short and long-distance movements have distinct requirements.

Recombinant viruses TMD_ArMV-EGFP and TMDR_ArMV-EGFP both exhibited a cell-to-cell movement (at least) as efficient as GFLV-EGFP (Figure 4). Furthermore, their inoculated leaves generated a positive DAS-ELISA signal and produced well-formed viral particles from which the three IC-RT-PCR fragments could easily be amplified (Figure 4, Figure 7, Figure S2). The biological properties of these recombinants again confirm that the three residues T258, M260, and D261 of region R4 and the residue R301 of region R5 are not involved in cell-to-cell movement of GFLV. Interestingly, the additional substitution of R301 by its ArMV equivalent in the recombinant virus TMDR_ArMV-EGFP led to a very potent systemic virus compared to TMD_ArMV-EGFP. Indeed, 100% of the plants inoculated with the TMDR_ArMV-EGFP transcripts showed symptoms and were DAS-ELISA positive in upper non-inoculated leaves, whereas only 63% of the plants inoculated with GFLV-EGFP and 31% of those inoculated with recombinant TMD_ArMV-EGFP showed systemic infection (Figure 4). Again, the progeny of each systemically infected plant was checked by IC-RT-PCR and sequencing: no reversion and no compensatory mutation were observed.

Thanks to our site-directed mutagenesis on the GFLV 2C^CP^ region R4 and residue R301, we succeeded in generating an infectious recombinant virus containing three residues of region R4 (and one in region R5) of ArMV origin, for which it was now possible to evaluate the impact on the virus transmission by its nematode vector *Xiphinema index*.

### 3.7. The Four Residues T258, M260, D261, and R301 are Critical for GFLV Transmission by X. index

**Figure 8:**
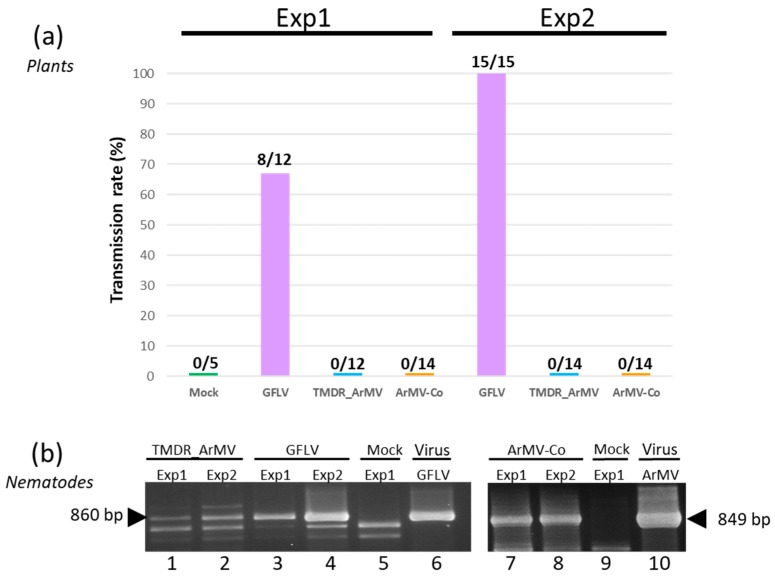
Involvement of the four residues T258, M260, D261 and R301 in GFLV transmission. (**a**) The transmission by *X. index* of wild type GFLV, chimera TMDR_ArMV, and natural ArMV-Co isolate was evaluated in two independent experiments. Transmission rates are expressed as the percentage of ELISA-positive bait plants. (**b**) RT-PCR detection of the viruses in *X. index* nematodes at the end of the AAP shows that nematodes had access to all tested viruses. Thirty nematodes exposed to source plants either infected with chimera TMDR_ArMV (lanes 1 and 2), synthetic GFLV (lanes 3 and 4), natural ArMV-Co (lanes 7 and 8), or mock inoculated plants (lanes 5 and 9) were randomly collected and tested by RT-PCR with GFLV (lanes 1 to 5) or ArMV (lanes 7 to 9) specific primers. Lanes 6 and 10 correspond to amplicons from purified GFLV and ArMV-Co, respectively. Nematodes from experiment 1 (lanes 1, 3 and 7) and 2 (lanes 2, 4, 8) were tested. The DNA products were analyzed by electrophoresis on 1% agarose gels.

The transmissibility of the recombinant virus TMDR_ArMV by *X. index* was evaluated in controlled conditions in the greenhouse and compared to the transmission of both the wild type GFLV deriving from the infectious clones (noted GFLV) and a natural ArMV isolate (ArMV-Co) as positive and negative controls, respectively. Two independent transmission experiments were performed with slightly different acquisition procedures (see Materials and Methods Section). Transmission was assessed by DAS-ELISA on a total of 26 to 28 bait plants (Figure 8). Like for ArMV, no transmission event was observed in the two transmission tests for the chimeric virus TMDR_ArMV, whereas GFLV was transmitted in both experiments to 67% and 100% bait plants (Figure 8). In both tests, we checked that nematodes had access to the virus during the AAP by RT-PCR performed on nematodes at the end of the AAP. DNA fragments of the expected size were amplified from nematodes that had fed on the roots of source plants infected with all viral constructs. Altogether, these results demonstrate that T258, M260, D261, and R301 constitute a new determinant of GFLV transmission by *X. index*.

## 4. Discussion

Of the five peptidic regions (R1 to R5) previously predicted to be exposed at the surface of the GFLV virions and potentially accessible for interactions, either with the nematode for specific transmission or with the plant for cell-to-cell and long-distance movement, region R4 was investigated in detail in this work. Through the use of a recombinant virus encoding EGFP, we could visualize the cell-to-cell spread of the infection and discriminate between a local site of infection produced by the wild type virus and a subliminal infection caused by a mutant impaired in capsid formation or stability.

Although the chimeric virus G4, where the 2C^CP^ subunit of GFLV contains region R4 of ArMV origin, is able to protect its genomic RNAs in an RNase protection assay, the recombinant virus G4-EGFP appeared to be restricted to small sites in inoculated leaves. We could show that this defective cell-to-cell movement was due to improper capsid formation rather than to the loss of interaction between the mutated capsid and the tubules, as previously hypothesized. EGFP visualization-assisted point mutagenesis was then performed to recover a mutant affected in region R4 of the 2C^CP^ protein, which is still capable of producing a systemic infection. To this end, we produced mutants where four amino acids (T258, M260, D261, and R301, the three first being located in region R4 and the last being part of region R5), were either changed into alanines or into their V258, N260, N261, and S301 counterparts in polyprotein P2-U of ArMV-S. The latter, named TMDR_ArMV, was fully infectious and was thus tested for transmission by *X. index*. The loss of transmission of this chimeric virus identifies these four residues as an additional important determinant of GFLV transmission.

**Figure 9:**
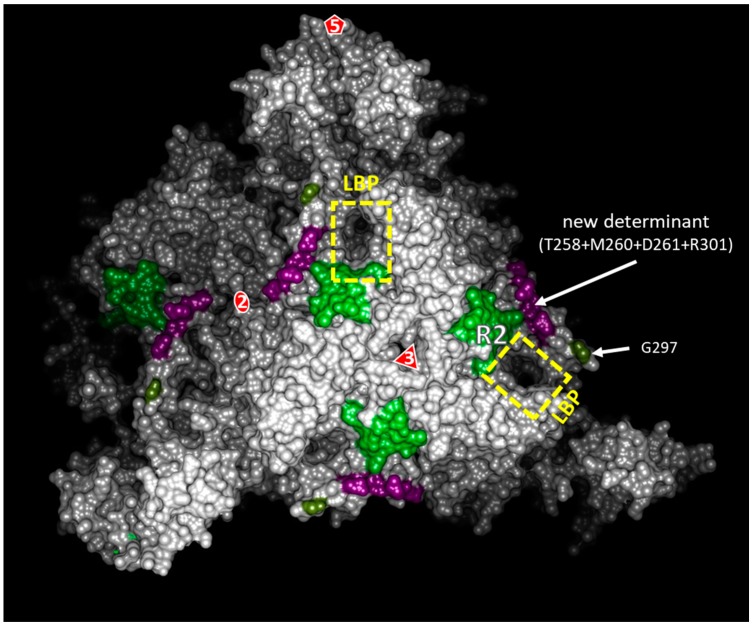
Surface view of a part of the GFLV viral particle, highlighting the hypothetical ligand-binding pocket and transmission determinants. This surface view is calculated from the GFLV-F13 atomic structure (PDB: 4V5T). The 2-fold, 3-fold, and 5-fold symmetry axes of the viral particle are indicated with a number and a red shape linked to the number. Two exemplars of the hypothetical ligand-binding pocket (LBP) are delineated by a yellow rectangle. R2 regions, G297 residues, and all copies of our new transmission determinant (T258 + M260 + D261 + R301) appearing on this surface view are colored in bright green, khaki, and lilac colors, respectively.

This new transmission determinant completes our map of the 2C^CP^ residues involved in GFLV transmission by *X. index* (Figure 9). Previously, a ligand-binding pocket (LBP) was proposed as the site of GFLV retention in the nematode’s mouthparts. This cavity is delineated by three loops, namely loops βB-βC, βG-βH, and βC’-βC’’ that contain regions R2, R3, and R5, respectively. The involvement of region R2 and of G297 of region R5 in the transmission of GFLV by *X. index* was demonstrated earlier [21,27], and led to the hypothesis that the whole cavity could constitute the binding site of the virus into the vector (LBP hypothesis) [21]. Here, we present additional evidence of the role of the LBP in vector transmission and more particularly of the βG-βH loop that contains the R301 residue. Whether the three amino acids of region R4 directly participate in GFLV transmission or whether they stabilize the capsid together with R301 remains to be determined.

The global positive charge of the LBP is expected to be of importance for the transmission specificity of GFLV since this cavity has the opposite charge on the 2C^CP^-U of ArMV-S [39]. Furthermore, the G297D substitution that negatively charges the LBP strongly impairs the transmission of the virus whereas the G297A modification preserves GFLV transmission [21]. In this respect, the residue R301 identified here is of particular interest, since along with residue R293, it takes part in the positive charge of the LBP [39]. Its replacement by a serine in the chimeric virus TMDR_ArMV greatly modifies the global charge of the pocket and could thus partly or totally account for the loss of transmission of this new chimera. To dissect the relative contribution of regions R4 and R5 to the transmission of the virus, the point mutant R301S should be constructed and the transmission of both this R301S and the TMD_ArMV chimera should be tested.

The G4 2C^CP^, although preserving the genomic RNAs from a ribonuclease treatment [27], presents a defect in capsid formation that accounts for the lack of systemic infection. It has to be noted that the swapping of both regions R2 and R4 in the G24 double recombinant led to the loss of RNA protection, whereas the single recombinants did protect their genomic RNAs from degradation [27]. This already indicates that at least one of the swapped regions likely modifies the capsid to some extent. It also suggests that RNA protection can be achieved by improperly assembled 2C^CP^ subunits, which are unsuited for efficient cell-to-cell movement and thus that an efficient cell-to-cell movement not only requires the presence of the 2C^CP^, but also its canonical assembly into a high-quality capsid.

The fact that G4-EGFP, which assemble abnormal capsids, still moves over a few cell layers argues against the involvement of the amino acids of region R4 as determinants of the specific capsid/tubule interaction as previously proposed. This is further confirmed by the cell-to-cell movement of the mutants targeting region R4 and/or residue R301 (mutants TMDR_Ala-EGFP, TMD_ArMV-EGFP, and TMDR_ArMV-EGFP). Similarly, the limited but indubitable cell-to-cell movement of G3-EGFP, despite its poor capsid formation, suggests that its residues are likely not involved in the specific transport of GFLV by its cognate tubules. Furthermore, the clear correlation between the low number of capsids observed in fixed tissues infected by the G3- and G4-EGFP recombinants and the inefficiency of these viruses to move over several cell layers, argues against the existence of an alternative, like a ribonucleoprotein complex, to the movement of the virus as virions in tubules, as proposed for other tubule-forming viruses like brome mosaic virus [40,41,42].

The mutant TMDR_Ala-EGFP, where the four amino acids T258, M260, D261, and R301 were replaced by four alanines, is also of very high interest. This mutant produces easily detectable capsids, encapsidates its genomic RNAs as deduced from IC-RT-PCR analyses, and moves cell-to-cell as well as the wild type GFLV-EGFP control. However, this locally effective infection is only systemic in 10% of the inoculated plants, highlighting that the requirements of the 2C^CP^ for cell-to-cell and long-distance movements differ, even for viruses with a tubule guided movement strategy, as previously reported for viruses using other movement strategies like tobacco etch virus [43], cucumber mosaic virus [44], or olive latent virus 1 [45]. These different requirements could concern the capsid conformation, which could be exposed to very different biochemical conditions in plasmodesmata containing tubules versus the phloem. Alternatively, the modifications introduced into the capsid could impair some interactions between the virus and the host that are required for the virus uptake into the phloem or the release from the phloem into apical leaves. A silencing suppressor activity has been proposed for a nepovirus CP [46] and one can also speculate that the TMDR_Ala-EGFP mutant could be impaired in systemic movement due to a reduced silencing suppressor activity of its 2C^CP^ protein. However, such a function has not been established for GFLV or ArMV 2C^CP^ proteins. Additionally, although this hypothesis cannot be ruled out, it is expected that such an impairment of silencing suppression would also have a drastic effect locally on the size of the infection foci [47]. A more detailed characterization of this GFLV mutant presenting a so far undescribed phenotype among nepoviruses would likely shed light on the mechanism(s) underlying systemic movement.

## 5. Conclusions

In this manuscript, we showed that a limited GFLV cell-to-cell movement is possible even when capsids are improperly formed, although, efficient tubule-guided cell-to-cell movement depends on the quality of the assembled particles. We also showed that cell-to-cell and long-distance virus movement can be uncoupled, as the requirements for these two movements differ, but both movements are critical for high virus accumulation in the roots and uptake by the nematode vector. Importantly, we identified a new viral determinant involved in the transmission of GFLV by its nematode vector, consisting of four residues in the 2CCP protein: T258, M260, D261 and R301. This new viral determinant is in close proximity to a positively charged cavity of the capsid, which strengthens the hypothesis that this cavity could constitute the ligand-binding pocket of GFLV in the nematode’s mouthparts. In the future, this work could be highly valuable to look for the GFLV receptor within the nematode.

## Supplementary Materials

The following are available online at https://www.mdpi.com/1999-4915/11/12/1146/s1. Figure S1: In planta ectopic expression of the TagRFP (TR) fused capsid proteins GFLV 2C^CP^:TR, TMDR_ArMV 2C^CP^:TR, G4 2C^CP^:TR and E264A 2C^CP^:TR; Figure S2: RNA encapsidation of GFLV viruses having mutations in the 2C^CP^ R4 region; Table S1: list of primers used for site-directed mutagenesis; Table S2: list of primers used to perform Immunocapture Reverse Transcription Polymerase Chain Reaction (IC-RT-PCR) and sequencing.
