# Supplementary material for: From a Movement-Deficient Grapevine Fanleaf Virus to the Identification of a New Viral Determinant of Nematode Transmission
Source: Viruses. 2019 Dec 11;11(12):1146. doi: 10.3390/v11121146 (PMC6950213; doi:10.3390/v11121146)
Supplement: Supplementary file 1 [file viruses-11-01146-s001.pdf]

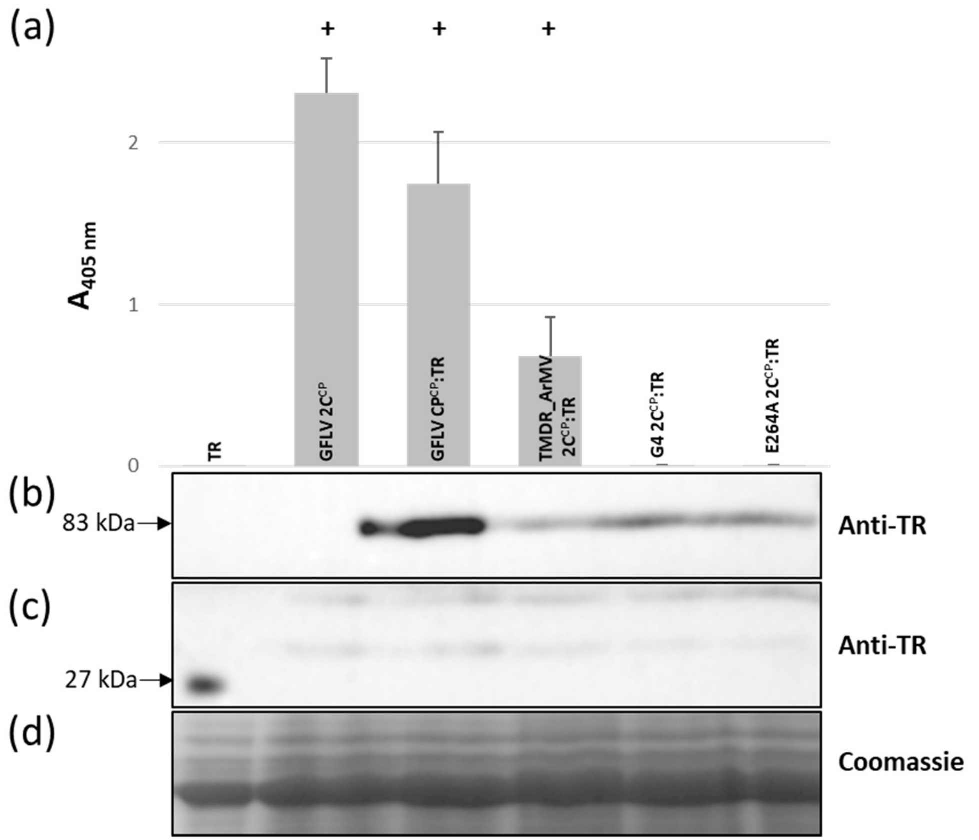

Figure S1. In planta ectopic expression of the TagRFP (TR) fused capsid proteins GFLV $2C^{CP}$:TR, TMDR_ArMV $2C^{CP}$:TR, G4 $2C^{CP}$:TR and E264A $2C^{CP}$:TR. GFLV $2C^{CP}$ and free TR are used as controls. Transient expression was obtained by agro infiltration. At 7 dpi, capsid formation was assessed by (**a**) GFLV DAS-ELISA and (**b**) protein expression was checked by western blot. (**a**) Bars represent the mean absorbance obtained with three different leaves for each condition. Error-bars correspond to 95 % confidence intervals. (**b**) Total proteins were separated by SDS-PAGE and analyzed by western blotting using anti-TR antibodies to perform detection of the $2C^{CP}$:TR fusion protein (83kDa) and the free TR protein (27 kDa). The membrane was imaged with the G:Box system (Syngene). (**c**) corresponds to the bottom part of the membrane to visualize the free TR protein. (**d**) Coomassie-blue stained PVDF membrane to validate equal loading. Black arrowheads indicate expected protein sizes.

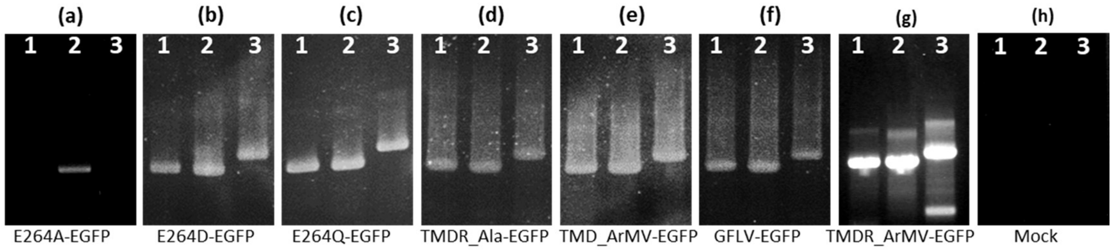

Figure S2. RNA encapsidation of GFLV viruses mutated in the 2C^{CP} R4 region. *C. quinoa* plants were inoculated with *in vitro* transcripts of GFLV RNA1 and an EGFP-expressing RNA2: (**f**) GFLV-EGFP (wild type control), (**a**) E264A-EGFP, (**b**) E264D-EGFP, (**c**) E264Q-EGFP, (**d**) TMDR_Ala-EGFP, (**e**) TMD_ArMV-EGFP, (**g**) TMDR_ArMV-EGFP or (**h**) were mock inoculated (negative control). At 7 dpi viral capsids from inoculated leaves were immunotrapped with anti-GFLV antibodies and encapsidated RNA2 were detected by RT-PCR. The F1 (1029 bp, lane 1), F2 (1009 bp, lane 2) and F3 (1180 bp, lane 3) fragments were analyzed on 1 % agarose gel and EtBr stained.

Table S1: list of primers used for site-directed mutagenesis

Name of the primer	Sequence (5' => 3')	Use of the primer
E264A	AACGGGACCACTATGGACTGGAATGCACTTTTTAAGTATC	production of the E264A-EGFP mutant
E264Arev	GATACTTAAAAAGTGCATTCCAGTCCATAGTGGTCCCGTT	production of the E264A-EGFP mutant
E264D	AACGGGACCACTATGGACTGGAATGACCTTTTTAAGTATC	production of the E264D-EGFP mutant
E264Drev	GATACTTAAAAAGGTCATTCCAGTCCATAGTGGTCCCGTT	production of the E264D-EGFP mutant
E264Q	AACGGGACCACTATGGACTGGAATCAACTTTTTAAGTATC	production of the E264Q-EGFP mutant
E264Qrev	GATACTTAAAAAGTTGATTCCAGTCCATAGTGGTCCCGTT	production of the E264Q-EGFP mutant
R301A	GCTTGCTGGTCAAAGTCAGGCAGACATGAGCTCTC	production of the TMDR_Ala-EGFP mutant
R301Arev	GAGAGCTCATGTCTGCCTGACTTTGACCAGCAAGC	production of the TMDR_Ala-EGFP mutant
R301S	GATTGCTTGCTGGTCAAAGTCAGAGCGACATGAG	production of the TMDR_ArMV-EGFP mutant
R301Srev	CTCATGTCGCTCTGACTTTGACCAGCAAGCAATC	production of the TMDR_ArMV-EGFP mutant
TMD_Ala	GTGGAACGGGGCAACTGCGGCCTGGAATGAACTTTTTAAGTATCCC	production of the TMD and TMDR_Ala-EGFP mutants
TMD_Alarev	GGGATACTTAAAAAGTTCATTCCAGGCCGCAGTTGCCCCGTTCCAC	production of the TMD and TMDR_Ala-EGFP mutants
TMD_ArMV	GTGGAACGGGGTCACTAACAACTGGAATGAACTTTTTAAG	production of the TMD and TMDR_ArMV-EGFP mutants
TMD_ArMVrev	CTTAAAAAGTTCATTCCAGTTGTTAGTGACCCCGTTCCAC	production of the TMD and TMDR_ArMV-EGFP mutants

Table S2: list of primers used to perform Immunocapture Reverse Transcription Polymerase Chain Reaction (IC-RT-PCR) and sequencing.

**Name of the primer**	**Sequence (5' => 3')**	**Use of the primer**
oligodT15	TTTTTTTTTTTTTTT	Retrotranscription
G34	CTWGATTTTAGGCTCAATGGTAT	PCR fragment F1 and sequencing
G35	ACTTATGGTGGATAAGCCAATG	PCR fragment F1 and sequencing
G36	GGTGTCCAGTATGAAAAGTGG	PCR fragment F2 and sequencing
G37	AAGAAACGAGAACCAATCTCAA	PCR fragment F2 and sequencing
432	ACTTGCCCTCCCATATTCTTTG	PCR fragment F3 and sequencing
G39	ATAAATTTGCAAAACAGTAAAAAGA	PCR fragment F3 and sequencing
